# Supplementary material for: Exogenous melatonin improves growth in hulless barley seedlings under cold stress by influencing the expression rhythms of circadian clock genes
Source: PeerJ. 2021 Jan 22;9:e10740. doi: 10.7717/peerj.10740 (PMC7831369; doi:10.7717/peerj.10740)
Supplement: Supplemental Information 1 [file peerj-09-10740-s001.docx]

**Table S1: The sequences of primers for qPCR.**

| Target genes in *Hordeum vulgare* L. | Orthologous genes in *Arabidopsis thaliana* | Sequence of upper primer for qPCR | Sequence of lower primer for qPCR | References |
| --- | --- | --- | --- | --- |
| *HvCCA1*  (N603242) | *AtCCA1*  (At2g46830) | 5’-CCAGCAGCAGACAGCAACATCC-3’ | 5’-CCGAAGCCGCAGCAACTGTAG-3’ | (Campoli et al. 2012) |
| *HvTOC1*  (HQ850268) | *AtTOC1*  (At5g61380) | 5’-TCCAGGGACGTTGAGTTGGTT-3’ | 5’-TTTTGAGCGGTTGGGGGTTG-3’ | (Deng et al. 2015) |
| *HvGI*  (AY740524) | *AtGI*  (At1g22770) | 5’-AGGCGAAATGGTAATGTTGC-3’ | 5’-CAGACATCTGCGTTTCAGGA-3’ | (Dunford et al. 2005) |
| *HvPRR73*  (AK376549) | *AtPRR7*  (At5g02810) | 5’-GCAACATTTCGGGGAAGCTG-3’ | 5’-TGCCATTTGAGCCCTGCTTT-3’ | (Campoli et al. 2012) |
| *HvPRR95*  (AK252005) | *AtPRR5*  (At5g24470) | 5’-TGCACGTTGAAATCCCCTCA-3’ | 5’-GATGCAACCCCTCCATGCTT-3’ | (Campoli et al. 2012) |
| *HvPRR59*  (AK361360) | *AtPRR9*  (At2g46790) | 5’-AGTGGGGTTTTGCCCTCACA-3’ | 5’-GCAGCACCAACAGGAATTGG-3’ | (Campoli et al. 2012) |
| *HvLUX*  (AK357505) | *AtLUX*  (At3g46640) | 5’-AATTCAGTCCACGGATGCTC-3’ | 5’-CTTCACTTCAGCTCCCCTTG-3’ | (Campoli et al. 2013) |
| *HvELF3*  (KY284997) | *AtELF3*  (At2g25930) | 5’-CCTACCGACAACAAGCAGAA-3’ | 5’-CATGAATTCCCCAGCTGTAG-3’ | (Xia et al. 2017) |
| *HvEF-1α*  (JN107538.1) | - | 5’-CCAACTTCACTGCCCAGGTCA-3’ | 5’-CACAGCAACCGTCTGCCTCAT-3’ | (Cai et al. 2018) |


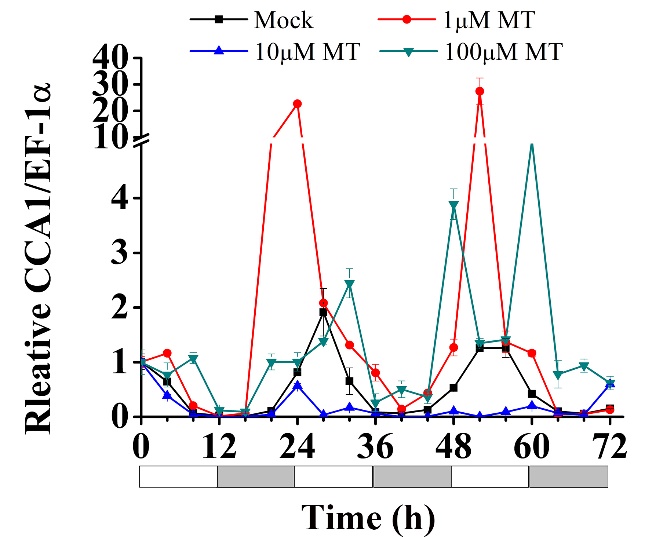


**Figure S1.** Expression of circadian clock gene *HvCCA1* in hulless barley seedlings treated with 1μM, 10μM, 100μM melatonin at 25 ℃. Plots of measured expression profiles of circadian clock genes for the 25 ℃ group (black lines), 1μM MT group (red lines), 10μM MT group (blue lines) and 100μM MT group (green lines) hulless barley seedlings. Total RNA for the analysis of the clock gene *HvCCA1* was extracted from 3 biological replicates. Average Cq values from 3 independent RT-qPCR amplifications were used to determine the relative expression levels of target genes after calibration with a reference gene, *EF-1α*. Error bars represent standard errors.


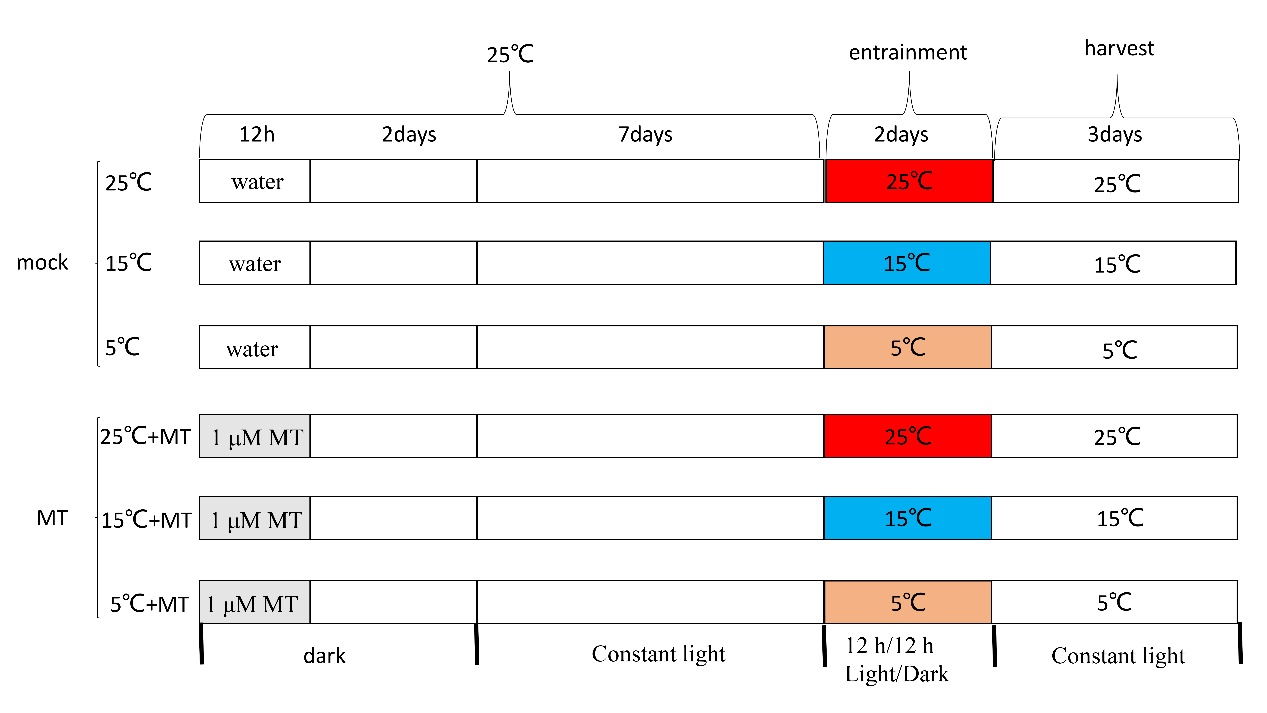


**Figure S2.** Overview of the treatments in experiment. Mock, the seeds were soaked in distilled water for 12 h in darkness at 25℃ before germination; MT, the seeds were soaked in 1 μM melatonin solution for 12h in darkness at 25℃ before germination.


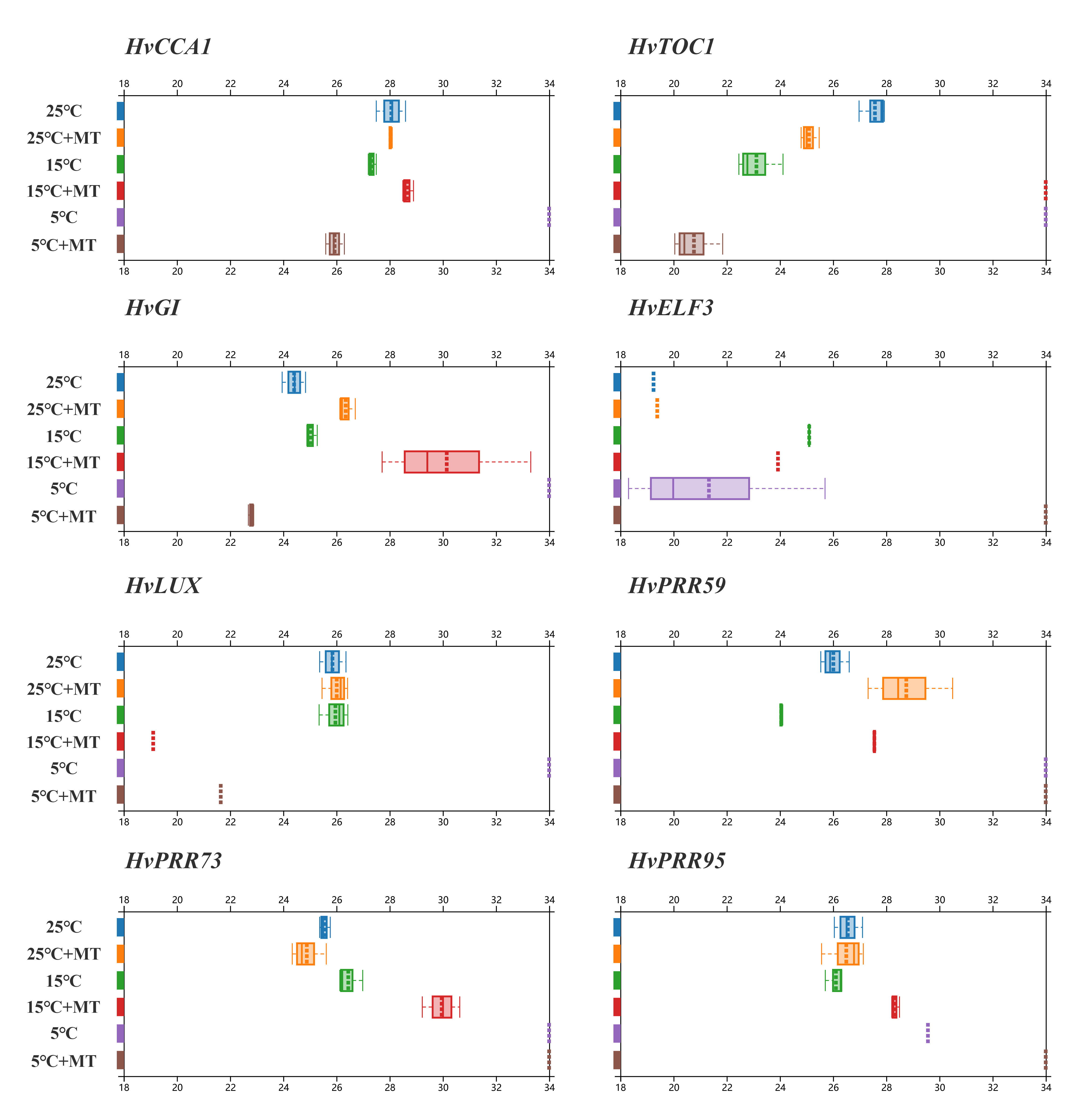


**Figure S3** Period lengths of clock genes circadian oscillation under different conditions in hulless barley seedlings. The results were analyzed by Spectrum Resampling through the online BioDare2 interface ([www.biodare.ed.ac.uk](http://www.biodare.ed.ac.uk)). The dashed line in the boxplot represents the average of the cycle lengths of the three biological replicates. Error bars represent standard errors. When the given rhythmic period length of a gene by BioDare2 is longer than 34 h or less than 18 h, it is screened to be an arrhythmic phenotype.


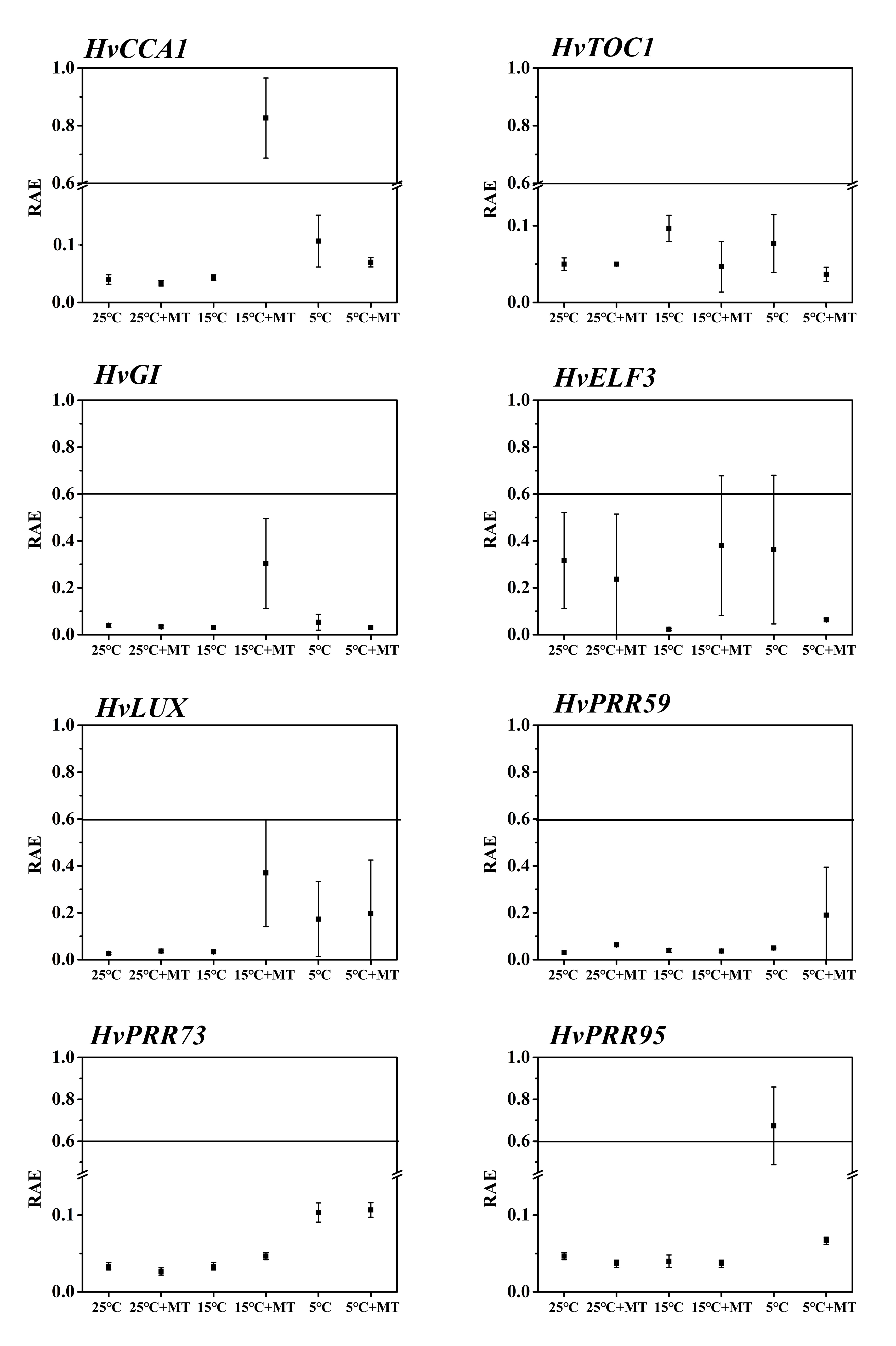


**Figure S4.** Relative amplitude error (RAE) analysis for clock genes circadian oscillation under different conditions in hulless barley seedlings. The results were analyzed by Spectrum Resampling through the online BioDare2 interface ([www.biodare.ed.ac.uk](http://www.biodare.ed.ac.uk)). When the RAE ≥ 0.6, it is generally considered that this group of periodic statistics is meaningless. Data indicate mean of three independent assays for every group. Error bars represent standard errors.

**Reference**

Cai J, Li P, Luo X, Chang T, Li J, Zhao Y, and Xu Y. 2018. Selection of appropriate reference genes for the detection of rhythmic gene expression via quantitative real-time PCR in Tibetan hulless barley. *PLoS ONE* 13(1): e0190559. https://doi.org/10.1371/journal.pone.0190559

Campoli C, Pankin A, Drosse B, Casao CM, Davis SJ, and von Korff M. 2013. HvLUX1 is a candidate gene underlying the early maturity 10 locus in barley: phylogeny, diversity, and interactions with the circadian clock and photoperiodic pathways. *New Phytologist* 199:1045-1059. https://doi.org/10.1111/nph.12346

Campoli C, Shtaya M, Davis SJ, and von Korff M. 2012. Expression conservation within the circadian clock of a monocot: natural variation at barley Ppd-H1 affects circadian expression of flowering time genes, but not clock orthologs. *BMC Plant Biology* 12:97. https://doi.org/10.1186/1471-2229-12-97

Deng W, Clausen J, Boden S, Oliver SN, Casao MC, Ford B, Anderssen RS, and Trevaskis B. 2015. Dawn and Dusk Set States of the Circadian Oscillator in Sprouting Barley (Hordeum vulgare) Seedlings. *PloS One* 10:e0129781. https://doi.org/10.1371/journal.pone.0129781

Dunford RP, Griffiths S, Christodoulou V, and Laurie DA. 2005. Characterisation of a barley (Hordeum vulgare L.) homologue of the Arabidopsis flowering time regulator GIGANTEA. *Theoretical and Applied Genetics* 110:925-931. 10.1007/s00122-004-1912-5

Xia T, Zhang L, Xu J, Wang L, Liu B, Hao M, Chang X, Zhang T, Li S, Zhang H, Liu D, and Shen Y. 2017. The alternative splicing of EAM8 contributes to early flowering and short-season adaptation in a landrace barley from the Qinghai-Tibetan Plateau. *Theoretical and Applied Genetics* 130:757-766. https://doi.org/10.1007/s00122-016-2848-2
